# Macrophage-Activating Lipopeptide-2 Requires Mal and PI3K for Efficient Induction of Heme Oxygenase-1

**DOI:** 10.1371/journal.pone.0103433

**Published:** 2014-07-31

**Authors:** Xiaoxing You, Liangzhuan Liu, Yanhua Zeng, Ranhui Li, Jun He, Xiaohua Ma, Chuanhao Jiang, Cuiming Zhu, Liesong Chen, Minjun Yu, Guangli Ou, Yimou Wu

**Affiliations:** 1 Institution of Pathogenic Biology, Medical College, University of South China, Hengyang City, P.R. China; 2 Department of Clinical Laboratory, Changsha Central Hospital, Chagnsha City, P.R. China; 3 Department of Clinical Laboratory, The Affiliated Nanhua Hospital of University of South China, Hengyang City, P.R. China; Chang Gung University, Taiwan

## Abstract

**Aims:**

This study is to investigate the mechanisms by which macrophage-activating lipopeptide-2 (MALP-2) induces heme oxygenase (HO)-1, a cytoprotective enzyme that catalyzes the degradation of heme, in human monocytes.

**Methods:**

Human monocytic THP-1 cells were cultured for transient transfection with plasmids and stimulation with MALP-2 for indicative time intervals. After incubation with MALP-2, cells were collected and disrupted, before being tested for promoter activity using luciferase assay. For analysis of proteins, immunoreactive bands were detected using an enhanced chemiluminescence Western blotting system, and the band intensity was measured by densitometryic analysis. For the detection of co-immunoprecipitation, SDS-PAGE was performed and the membranes were probed using respective antibodies. To investigate the cellular localization of NF-E2-related factor 2 (Nrf2), cells underwent immunofluorescence staining and confocal microscopy, and were analyzed using electrophoretic mobility shift assay.

**Results:**

MALP-2-induced HO-1 expression and promoter activity were abrogated by transfection with dominant negative (DN) plasmids of TLR2 and TLR6, or their neutralizing antibodies. However, inhibition of MyD88 or transfection with the DN-MyD88 was insufficient to attenuate HO-1 expression. In contrast, mutation or silencing of MyD88 adapter-like (Mal) by DN-Mal or siRNA almost completely blocked HO-1 induction. Btk, c-Src and PI3K were also involved in MALP-2-induced HO-1 expression, as revealed by specific inhibitors LFM-A13, PP1 and LY294002, or by transfection with siRNA of c-Src. MALP-2-induced activation of PI3K was attenuated by transfection with DN mutant of Mal, and by pretreatment with LFM-A13 or PP1. Furthermore, MALP-2 stimulated the translocation of Nrf2 from the cytosol to the nucleus and Nrf2 binding to the ARE site in the HO-1 promoter, which could also be inhibited by pretreatment with a PI3K inhibitor, LY294002.

**Conclusions:**

These results indicated that MALP-2 required TLR2/6, Btk, Mal and c-Src to activate PI3K, which in turn initiated the activation of Nrf2 for efficient HO-1 induction.

## Introduction

Mycoplasma is a kind of the smallest cellular organisms that are capable of self-replicating and persist as obligate extracellular parasites [1], [2]. Mycoplasma infects nearly 2 million people yearly [3], and is responsible for up to 40% of the community-acquired pneumonia diagnosed in children. Strong clinical associations also exist between some mycoplasmas and male nongonococcal urethritis, and more recently, genital infections have also been correlated with lower and upper reproductive tract inflammation in women [4]. During mycoplasma infection, invading pathogens interact with the local environment. As a result, inflammatory cells are activated and secrete a spectrum of cytokines and chemokines [5], [6]. These cytokines consist of a complicated synergetic or antistatic network and have been implicated in many disordered inflammatory diseases [7], [8].

The most common bacterial component implicated in the initiation of the inflammatory response by mycoplasma is their membrane-bound lipoproteins [9], [10]. Macrophage-activating lipopeptide-2 (MALP-2), a synthetic molecular entity originally derived from *Mycoplasma fermentans*, shares many inflammatory properties of membrane lipoproteins in almost all of the mycoplasmal genera and thus is widely used as a candidate of inflammagen for mycoplasma studies [11]–[14]. Mycoplasma membrane lipoprotein or MALP-2 is mainly recognized by Toll-like receptor (TLR)2 and TLR6 [15], which were expressed on the cell surface of the innate immune cells [16]. After recognition, TLR2 and TLR6 undergo conformational changes that allow them to engage intracellular adapter molecules, such as myeloid differentiation primary response 88 (MyD88) and MyD88 adapter-like (Mal), also known as Toll/interleukin-1 receptor (TIR) domain-containing adapter protein (TIRAP), to the receptors and thus activate canonical IκB kinase and various mitogen-activated protein kinases (MAPKs) to increases the transcriptional activity of nuclear factor (NF)-κB and activator protein 1 (AP-1) [12], [17]. Activated TLR signaling results in the production of various inflammatory molecules such as inducible nitric oxide synthase, cyclooxygenase-2, tumor necrosis factor (TNF)-α and interleukin (IL)-1β to control and eliminate the infection by leukocyte recruitment and inflammation [11], [18].

The inflammatory response triggered by membrane lipoprotein or MALP-2 is predominantly controlled by monocytes or macrophages that produce various pro-inflammatory cytokines or mediators [19]. Inflammatory response is essential for constraining the infection, therefore it should be tightly regulated to homeostasis, or else, the inflammatory response can become excessive and may progress into unchecked [20], [21]. In this sense, there must be some intrinsic or adaptive mechanisms to protect against dysfunctional inflammation during infection. An increasing number of reports have indicated that the hosts have developed sophisticated negative mechanism to regulate the multiple layers of inflammatory response [20], [22], [23]. Indeed, some cell wall components of bacteria, such as lipoteichoic acids and lipopolysaccharides, were reported to activate the basic leucine zipper transcription factor NF-E2-related factor 2 (Nrf2), a key factor involved in antioxidant protein expression in human tracheal smooth muscle cells and monocytes [24]. Activation of Nrf2 induces the expression of various phase 2 detoxification genes, such as glutamate-cysteine ligase catalytic subunit, glutathione reductase, heme oxygenase-1 (HO-1) and NAD(P)H:quinone oxidoreductase 1 (NQO1), to protect against the deleterious effects of inflammatory actions [25], [26]. In monocytes, Nrf2-induced HO-1 and NQO1 expressions inhibit excessive proinflammatory cytokine secretion in response to lipopolysaccharides [27]. In a mouse model of experimental *Toxoplasma gondii* infection, pharmacological induction of HO-1 expression decreased parasite replication in lungs and small intestine of infected C57BL/6 mice [28]. Additionally, inhibition of HO-1 expression by a Bruton’s tyrosine kinase (Btk) inhibitor LFM-A13 significantly increased the sensitivity to heme induced cell toxicity [29]. Moreover, Lee et al. demonstrated that HO-1 functions as a suppressor of TNF-α signaling, not only by inhibiting the expression of adhesion molecules and generation of IL-6, but also by diminishing intracellular reactive oxygen species production and NF-κB activation [30]. Taken together, these studies suggest that HO-1 plays a crucial role in modulating the immune system. In our previous study, we have demonstrated that MALP-2 could also induce the expression of HO-1 in human monocytes via Nrf2 activation [31]. However, the regulatory mechanism remains to be elucidated.

In light of the importance of HO-1 in maintaining of the homeostasis under infection and oxidase stress condition, a considerable work have been done to investigate the signaling pathways involved in the regulation of HO-1 expression [24], [25]. Mal, which is essential for TLR2 signaling, was originally presumed only as a bridge adaptor to recruit MyD88 molecules to the activated TLR2 dimer on the plasma membrane. However, recent studies have indicated that Mal also has its own signaling pathways. For example, Mal contains several functional motifs such as TNF receptor-associated factor 6 (TRAF6)-binding motif, and mutations in this motif result in the inhibition of TLR2- and TLR4-mediated activation of NF-κB [32]. In addition, Mal can be phosphorylated by Btk, and cleaved by caspase-1 to modulate TLR2 and TLR4 signaling [33], [34]. Furthermore, Mal displays a great inhibitory role for TLR3 signaling to c-Jun N-terminal kinase (JNK) and IL-6 induction [35], but its role in mediating HO-1 expression is still unknown. Btk and c-Src, two non-receptor tyrosine protein kinases, have been shown to play multiple roles in macrophage-mediated innate immunity [36], [37]. Activated Btk and c-Src could phosphorylate their downstream of kinases, such as phosphatidylinositol-4,5-bisphosphate 3-kinase (PI3K)/Akt [38]. PI3K/Akt activation up-regulates HO-1 gene expression, and the protective effects of this signaling cascade might be linked to the salutary effects of HO-1 [39]. In addition, there is also evidence that PI3K and Mal are functionally linked to induce phosphatidylinositol (3,4,5)-triphosphate (PIP3) formation at the leading edge of macrophages [40]. On the other hand, PI3K/Akt-mediated Nrf2 activation has also been reported [41].

Despite the recognition of the importance of HO-1 in immune modulation, the upstream events after mycoplasma lipoprotein stimulation have not been addressed. Previously, Lee and colleagues reported that lipoteichoic acids induce tracheal smooth muscle cells expression of HO-1 via a TLR2/MyD88/c-Src/NADPH oxidase/Nrf2 pathway [24]. However, it was not clear whether this event has any impact on MALP-2-induced HO-1 expression. In the current study, we report that MyD88 is not necessary for HO-1 induction after MALP-2 stimulation, but is strongly associated with Mal to mediate PI3K activation, which ultimately leads to the activation of Nrf2 and then induces HO-1 expression. These results represent the first evidence, to our knowledge, for the importance of Mal in regulating HO-1 expression, suggesting a new negatively regulatory mechanism for inflammatory response.

## Materials and Methods

### Reagents

MALP-2 was purchased from Enzo Lifesciences (ALX-162-027-C050; Plymouth Meeting, PA). Anti-HO-1, anti-phosphorylated and anti-total c-Src, anti- phosphorylated Btk, anti-phosphorylated Akt and anti-total Akt antibodies were products of Cell Signaling Technology (Beverly, MA). Anti-total Btk and anti-Mal were from Abcam (Cambridge, MA). Anti-MyD88, anti-p85α and anti-Nrf2 antibodies were purchased from Santa Cruz (Santa Cruz, CA). Cy3-conjugated goat anti-rabbit IgG antibody was purchased from Invitrogen (Frederick, MD). Anti-TLR2 and anti-TLR6 neutralizing antibodies, dominant negative (DN) plasmids DN-TLR2 and DN-TLR6, and DN-Mal and DN-MyD88 were products of InvivoGen (San Diego, CA). Protein phosphatase 1 (PP1) was from Enzo Lifesciences (Plymouth Meeting, PA). LFM-A13 and LY294002 were obtained from Calbiochem (Darmstadt, Germany) and Sigma-Aldrich (St. Louis, MO), respectively. Complete protease inhibitor cocktail was obtained from Roche Applied Science (Mannheim, Germany).

### Cell culture

Human monocytic THP-1 cells were purchased from American Type Culture Collection and cultured in RPMI 1640 medium (Invitrogen, USA) supplemented with 10% fetal bovine serum, 2 mM L-glutamine, 100 µg/ml penicillin, and 100 µg/ml streptomycin. Cells were maintained in a humidified atmosphere at 37°C and 5% CO_2_. For stimulation experiments, THP-1 cells were seeded in serum-free medium in 6-well plates (1×10^6^/well) and allowed to cultivate overnight. The cells were stimulated with 5 ng/mL MALP-2 for indicative time intervals.

### Luciferase activity assay

A 3292-bp (−3106 to +186) segment from the 5′-promoter region of the HO-1 gene was cloned as described previously [24]. Briefly, a 3.29-kb segment at the 5′-flanking region of the human HO-1 gene was amplified by polymerase chain reaction (PCR) using specific primers from the human HO-1 gene (NC_018933.1): 5′CCGAGCTCGAGAACAGTTAGAAAAGAAAGC 3′ (forward/SacI) and 5′GGCTCGAGACAGGCAGGATCAGAACCCCG 3′ (reverse/Xho1). The pGL3-Basic vector, containing a polyadenylation signal upstream from the luciferase gene, was used to construct the expression vectors by subcloning PCR-amplified DNA of the HO-1 promoter into the SacI/Xho1 site of this vector. The PCR products (pGL3-HO-1) were confirmed by electrophoresis and sequencing. HO-luc plasmid and β-gal was transiently transfected into THP-1 cells by using FuGENE 6 reagent (Roche, Switzerland) according to the manufacturer’s instructions. After incubation with MALP-2, cells were collected and disrupted, and were tested for luciferase activity using the luciferase assay system (Promega, USA). Firefly luciferase activities were standardized for β-gal activity.

### Transient transfection

Commercially available small interfering RNA (siRNA) of Smartpool siRNA for human Mal, MyD88 and c-Src, and negative control non-targeting siRNA were purchased from Dharmacon (Thermo Scientific, USA). THP-1 cells (5×10^5^ cells/well) were transfected with siRNA using Lipofectamine 2000 (Invitrogen, USA) for 18 h. Transfection of the DN plasmid of TLR2, TLR6, MyD88 and Mal were performed using FuGENE 6 reagent (Roche, Switzerland) according to the manufacturer’s instruction. A total of 4×10^5^ THP-1 cells were transfected with 0.5 µg DN-TLR2, DN-TLR6, DN-MyD88 or DN-MyD88 expressing plasmid. After 20 h of transfection, the cells were incubated with indicated reagents for further experiments.

### Western blotting

Cell lysates were prepared using a NE-PER nuclear and cytoplasmic extraction reagents according to the manufacturer’s instructions (Pierce, USA). Samples were denatured, and equal amounts of protein were subjected to SDS-PAGE, and then transferred to nitrocellulose membrane. The membranes were blocked with 1% bovine serum albumin at room temperature for 1 h and incubated with specific indicated antibodies for 1 h, and then the membranes were incubated with the second antibodies for another 1 h. The immunoreactive bands were detected using an enhanced chemiluminescence Western blotting system (Thermo Scientific, USA), and the band intensity was measured by densitometric analysis using Image J software.

### Co-immunoprecipitation assay

Cell lysates containing 1 mg protein were incubated first with 2 µg of the respective antibodies at 4°C for 24 h, and then additionally with protein A/G beads and mixed for 24 h at 4°C. Beads were collected and washed three times with lysis buffer. Following washing, loading buffer was added and subjected to SDS-PAGE, and then the membranes were probed using anti-Btk, anti-Mal, anti-MyD88, anti-c-Src, or anti-p85α antibodies.

### Immunofluorescence staining

THP-1 cells were deposited onto glass coverslips by using a cytocentrifuge (StatSpin CytoFuge 12, Iris Sample Processing, Inc., USA), and then fixed with 3.5% paraformaldehyde in phosphate buffered saline (PBS) for 10 min at room temperature and permeabilized with 100% MeOH for 10 min. To investigate the cellular localization of Nrf2, cells were treated with a polyclonal antibody against Nrf2 for 2 h. After extensive washing with PBS, cells were incubated with a secondary cy3-conjugated goat-anti-rabbit antibody diluted at 1∶1000 in PBS for 1 h at room temperature. Nuclei were stained with 1 µg/mL of 4′-6-diamidino-2-phenylindole, and then analyzed by confocal microscopy using a Zeiss LSM 710 Meta microscope (Germany).

### Electrophoretic mobility shift assay (EMSA)

Nuclear protein extracts were prepared before EMSA. The oligonucleotide probe containing the human Nrf2 binding sequence, 5′-TGGGGAACCTGTGCTGAGTCACTGGAG-3′, was manufactured and biotinylated using a biotin 3′-end DNA labeling kit following manufacturer’s instructions (Thermo Scientific, USA). For supershift studies, nuclear extracts from MALP-2-treated THP-1 cells were preincubated with 1 µg Nrf2 antibody for 2 h. The reaction complexes were resolved on 5% acrylamide gel and transferred to positively charged Biodyne B nylon membrane. The membrane was cross-linked under UV light (312 nm) for 15 min. Complexes were detected using a chemiluminescent nucleic acid detection module following manufacturer’s instructions (Thermo Scientific, USA).

### Statistical analysis

Data are expressed as means ± SEM of triplicate determinations and relative to untreated cells. Student’s t-test was performed for comparison between two groups. P<0.05 was considered statistically significant. For Western blotting, EMSA and immunofluorescence experiments, data are representative of at least three independent experiments.

## Results

### MALP-2-induced HO-1 expression is mediated by TLR2 or TLR6

**Figure 1:**
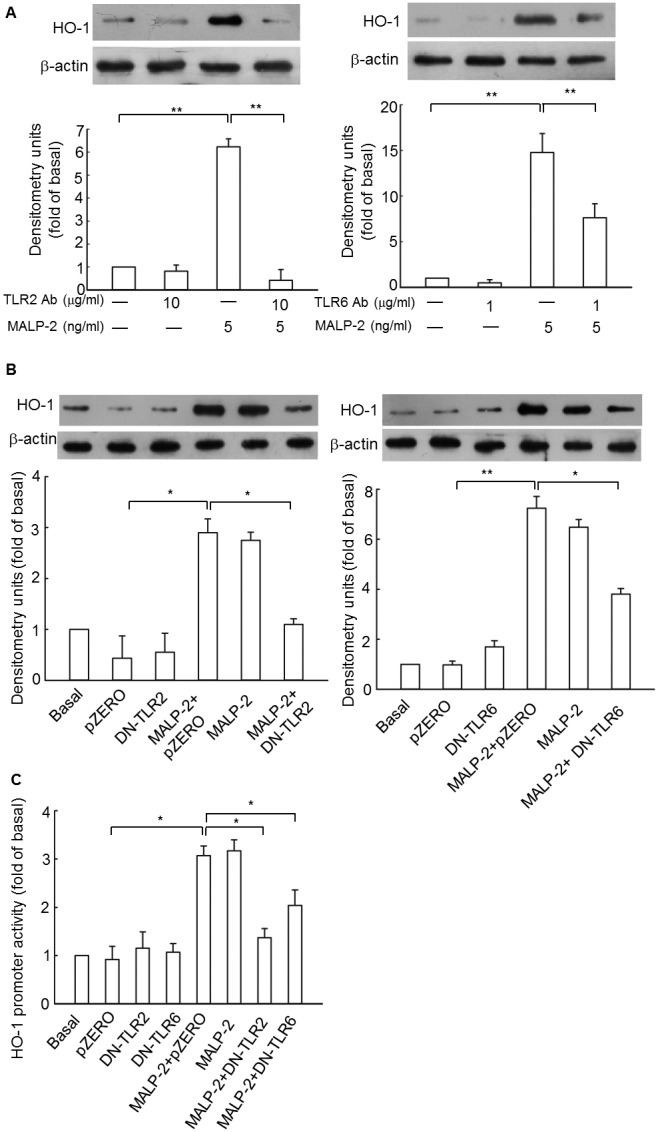
HO-1 expression in response to MALP-2 is dependent on TLR2 and TLR6. **A.** THP-1 cells were preincubated with anti-TLR2 (10 µg/ml) or anti-TLR6 (1 µg/ml) for 1 h, and then stimulated with MALP-2 (5 ng/ml) for 16 h. HO-1 protein expression was analyzed by Western blot, and then densitometric analysis was performed after normalization with β-actin protein levels. **B.** Cells were transiently transfected with a dominant negative (DN)-TLR2 or DN-TLR6. After 20 h of transfection, cells were incubated with 5 ng/ml of MALP-2 for another 16 h. The cell lysates were prepared and separated by SDS-PAGE for Western blot analysis of HO-1. **C.** Cells were cotransfected with a DN-TLR2 or DN-TLR6 and HO-1-luc reporter gene, and then stimulated with MALP-2 (5 ng/ml) for 8 h. The luciferase activity derived from HO-1 activation was normalized the transfection efficiency with β-gal, and pZERO was used as a control vector. Data shown represent results from three independent experiments (means ± SEM). *, P<0.05 and **, P<0.01 for significant difference between compared groups.

To explore whether MALP-2-induced HO-1 expression was mediated through TLR2 or TLR6, THP-1 cells were pretreated with the functional neutralizing anti-TLR2 (10 µg/ml) or anti-TLR6 (1 µg/ml) antibodies for 1 h, and then stimulated with 5 µg/ml MALP-2 for another 16 h. As expected, MALP-2-induced HO-1 expression was inhibited by pretreatment with anti-TLR2 or anti-TLR6 neutralizing antibodies, indicating the involvement of TLR2/6 in these responses (Fig. 1A, Fig. S1). This hypothesis was further supported by transfection of DN plasmids of TLR2 or TLR6 (Fig. 1B). Moreover, mutation of TLR2 or TLR6 also inhibited HO-1 promoter activity induced by MALP-2 (Fig. 1C). These results demonstrated that MALP-2-induced HO-1 expression was mediated by TLR2 or TLR6.

### c-Src participates in MALP-2-induced HO-1 expression

**Figure 2:**
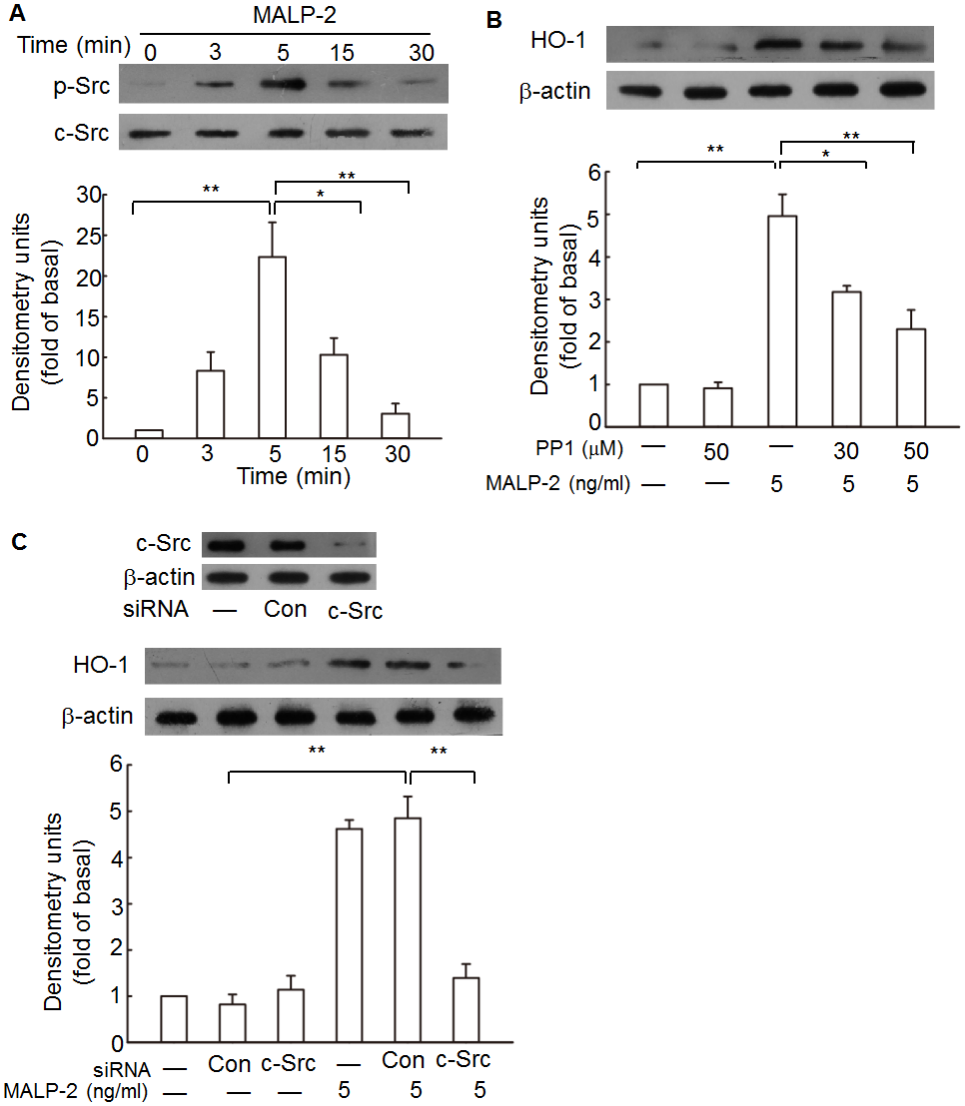
MALP-2-stimulation induces c-Src phosphorylation and HO-1 expression. **A.** THP-1 cells were stimulated with MALP-2 (5 ng/ml) for the indicated time points and lysed. The lysates were analyzed by Western blotting with a phosphorylated (p)-c-Src antibody (upper panel) or total c-Src antibody (lower panel). The results of a representative experiment are shown, and densitometric quantification of relative protein levels of p-c-Src from three experiments are presented under the panels. **B.** Cells were preincubated with PP1 for 1 h prior to stimulation with MALP-2. Cell lysates were prepared and HO-1 proteins were detected by Western blotting. **C.** Cells were transfected with c-Src specific siRNA or control (con) siRNA prior to 5.0 ng/ml MALP-2 treatment. Total c-Src and HO-1 protein expression levels were detected by Western blotting. Results shown are representative of at least three separate experiments. **, P<0.01 for significant difference between compared groups.

c-Src has been known to play critical roles in the progression of cancer and involved in the inflammation-related signaling pathways [30]. c-Src is activated through transient intermediates. It first becomes tyrosine dephosphorylation in residue 527 in the activation loop of the protein and further activation is achieved by autophosphorylation at the tyrosine residue 416 [42]. Thus, phosphorylation of the c-Src at Tyr416 site is a prerequisite for c-Src completely activation. To test whether the expression of HO-1 was mediated by c-Src, an anti-phospho-c-Src antibody at Tyr416 was used. The results showed that the increase in tyrosine phosphorylation of c-Src was detected within 5 min after MALP-2 stimulation (Fig. 2A, Fig. S2). In addition, pretreatment with a c-Src inhibitor PP1 significantly attenuated MALP-2-induced HO-1 expression in a concentration-dependent manner (Fig. 2B). Furthermore, transfection of THP-1 cells with c-Src siRNA significantly down-regulated c-Src and subsequently led to a decrease of HO-1 protein expression in response to MALP-2 (Fig. 2C). These results suggested that c-Src activation was involved in MALP-2-induced HO-1 expression.

### Btk is involved in MALP-2-induced HO-1 expression

**Figure 3:**
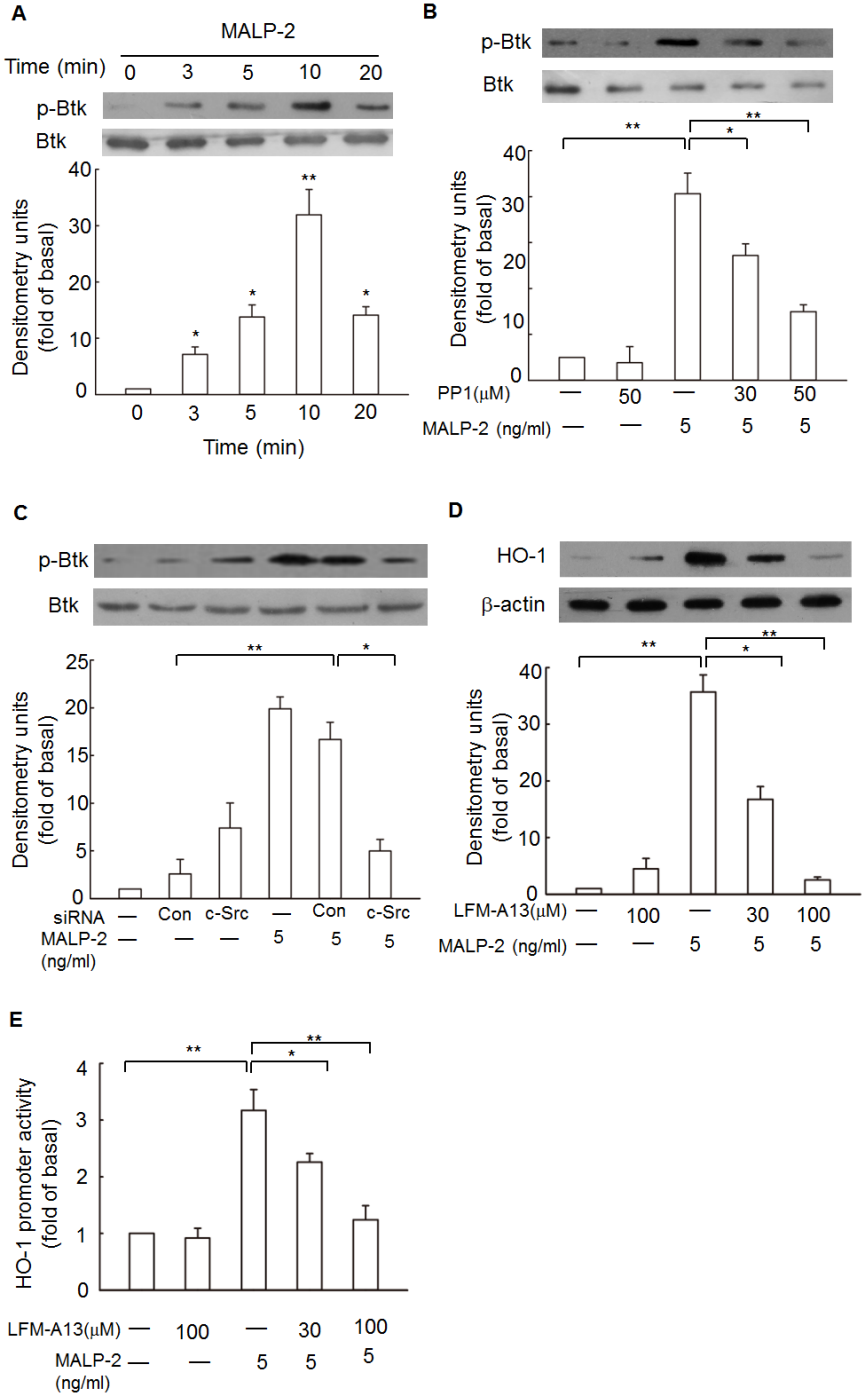
MALP-2 induces HO-1 expression in a Btk-dependent manner. **A.** THP-1 cells were stimulated with MALP-2 for the indicated time intervals, the cell lysates were subjected to Western blotting using an anti-phospho-Btk at Tyr223. *, P<0.05 and **, P<0.01 as compared with the basal level. **B.** Cells were pretreated with or without PP1 for 1 h, and then incubated in the absence or presence of 5 ng/ml MALP-2 for 10 min. Cell fractions were prepared and subjected to Western blotting analysis with an anti- phospho-Btk antibody. Data shown represents three experiments (means ± SEM). **C.** Cells were transfected with siRNA of c-Src, and then treated with MALP-2 for 10 min. Phosphorylated Btk was detected by Western blotting. Results shown are representative of at least three separate experiments. **D.** THP-1 cells were pretreated with a Btk inhibitor FLM-A13 (30 µM and 100 µM) for 1 h, and incubation was continued with MALP-2 (5 ng/ml) for another 16 h. Samples were subjected to Western blotting for the detection of HO-1 expression. Densitometry analysis was performed on at least three Western blots quantified by scanning densitometry. **E.** Cells were transiently transfected with HO-1-luc reporter gene, and then pretreated with LFM-A13. After incubation for 1 h, cells were stimulated with MALP-2 (5 ng/ml) for 8 h. The luciferase activity derived from HO-1 activation was normalized to the transfection efficiency with β-gal. Data represent means ± SEM from at least three independent experiments. *, P<0.05 and **, P<0.01 for significant difference between compared groups.

Btk is a non-receptor tyrosine kinase, and have been implicated in a double shift to control immune response [43]. To investigate its role in MALP-2 induced signaling, THP-1 cells were stimulated with MALP-2 for various lengths of time, and endogenous Btk was assessed for phosphorylation in the tyrosine residues at Tyr223, which was necessary for full activation of Btk [37]. Btk from stimulated cells exhibited increased Tyr223 phosphorylation within 10 min of stimulation using a phospho-Tyr223 antibody (Fig. 3A, Fig. S3). This finding supports a role for Btk in signaling via the TLR2,6 molecules. Previous data indicated that Src family kinases including c-Src regulated Btk activation [43], [44]. Therefore, activation of Btk was expected to be a consequence of c-Src activation in MALP-2-treated cells. To test this possibility, a temporal analysis of Btk activation was performed in THP-1 cells treated with the c-Src inhibitor PP1. Pretreatment of THP-1 cells with PP1 blocked MALP-2-induced Btk phosphorylation (Fig. 3B). To rule out the nonspecific effects of pharmacological inhibitors of c-Src, the effect of siRNA-mediated Btk phosphorylation was analyzed. Transfection of THP-1 with c-Src-specific but not scrambled control siRNA blocked MALP-2-induced Btk phosphorylation (Fig. 3C). These data therefore suggest that c-Src acts upstream of Btk in the signaling cascade induced in THP-1 by MALP-2.

To further determine whether MALP-2-induced HO-1 expression required Btk, a specific pharmacological Btk inhibitor LFM-A13 was used. Pretreatment of cells with LFM-A13 significantly inhibited the up-regulation of HO-1 protein in response to MALP-2 (Fig. 3D). Moreover, exposure to LFM-A13 also decreased HO-1 promoter activity in response to MALP-2 in a dose-dependent manner (Fig. 3E). These results indicated that Btk was involved in HO-1 gene activation.

### Dominant negative plasmid and siRNA of MyD88 are neither able to significantly attenuate HO-1 expression

**Figure 4:**
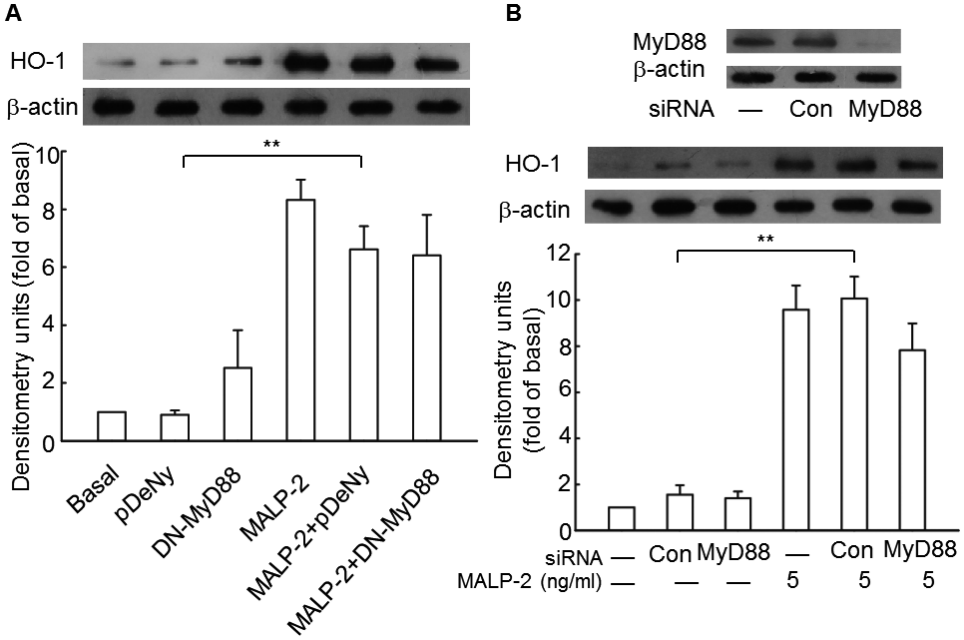
MyD88 does not play a central role in MALP-2-induced HO-1 expression. **A.** THP-1 cells were transiently transfected with a dominant negative plasmid encoding MyD88 (DN-MyD88) or empty vector (pDeNy, lane 2). Cell lysates were prepared, and samples were immunoblotted with an anti-HO-1 antibody. **B.** Cells were transfected with MyD88 siNRA or control siRNA, and then treated with MALP-2 (5 ng/ml) for 16 h. Expression of MyD88 and HO-1 were analyzed by Western blotting. All values are expressed as means ± SEM obtained from three independent experiments. **, P<0.01 for significant difference between compared groups.

Upon activation of TLR, MyD88 is recruited to TLR domains and links TLR with the downstream intracellular signaling cascades [45]. To determine whether MyD88 plays an important role in HO-1 expression induced by MALP-2, we transfected THP-1 cells with either a dominant negative plasmid, pDeNy-hMyD88, which is a truncated form of MyD88 that contains the C-terminal TIR domain but lacks the death domain, or MyD88 siRNA to silent its expression. The results showed that functional mutation of MyD88 failed to reduce HO-1 expression significantly (Fig. 4A, Fig. S4), and transfection with MyD88 siRNA did not significantly block HO-1 expression. These data suggested that MyD88 was not a prerequisite for efficient HO-1 induction by MALP-2.

### Mal plays a greater role than MyD88 in HO-1 induction by MALP-2

**Figure 5:**
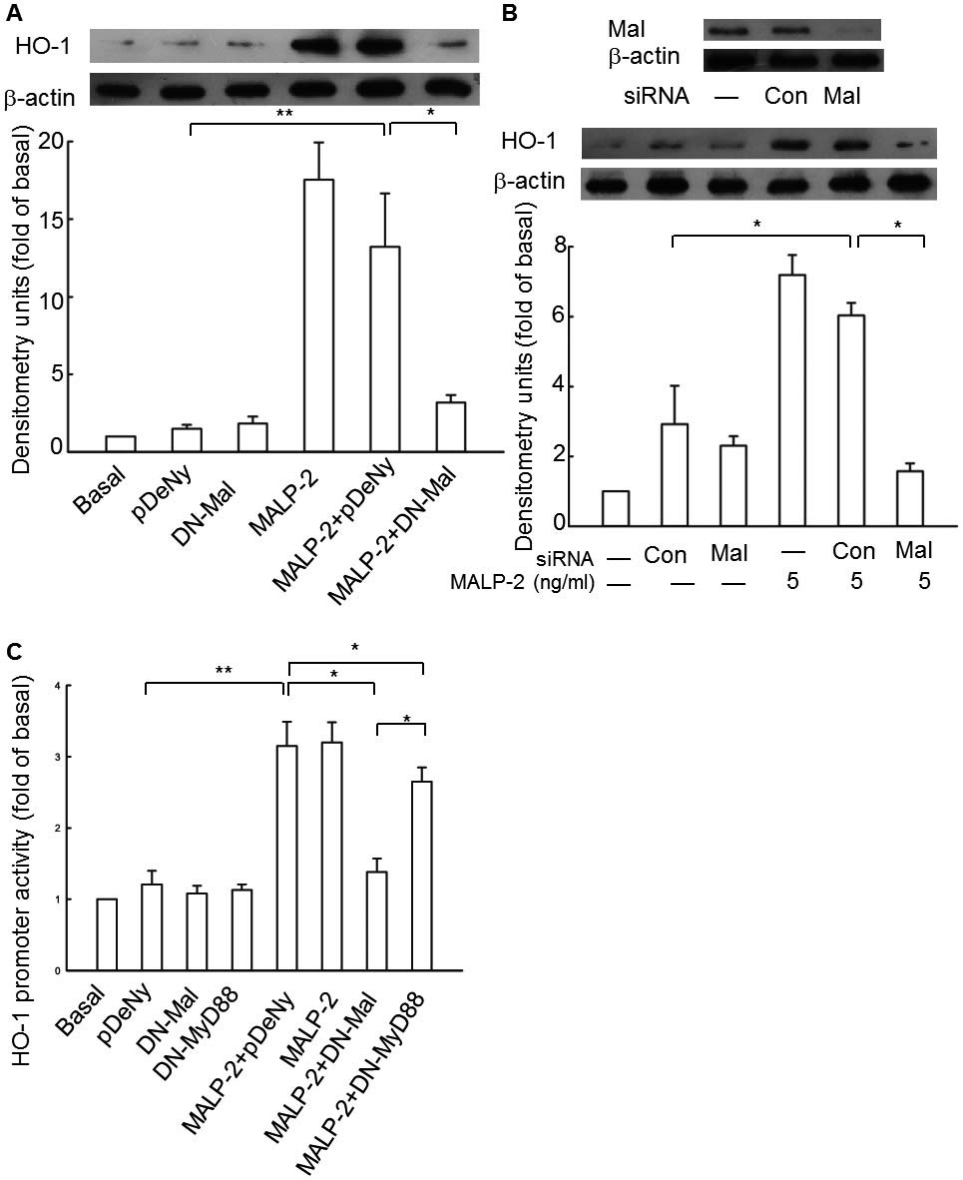
Mal is essential for the expression of HO-1 induced by MALP-2. **A.** Cells were transiently transfected with a dominant negative plasmid encoding Mal (DN-Mal) or empty vector (pDeNy, lane 2). Cell lysates were prepared, and samples were immunoblotted with an anti-HO-1 antibody. **B.** Cells were transfected with Mal siNRA or control siRNA, and then treated with MALP-2 (5 ng/ml) for 16 h. Expression of Mal and HO-1 were analyzed by Western blot. **C.** Cells were cotransfected with a DN-MyD88 or DN-Mal and HO-1-luc reporter gene, and then treated with MALP-2 for 8 h. The luciferase activity derived from HO-1 activation was normalized to the transfection efficiency with β-gal, and pDeNy was used as a control vector. All values are expressed as means ± SEM obtained from three independent experiments. *, P<0.05 and **, P<0.01 for significant difference between compared groups.

Mal is often described as a bridging adaptor due to its primary function of recruiting cytosolic MyD88 to interact with the activated TIR domains of TLRs dimers at the cell membrane. However, updated data indicate that Mal is no just as a bridge to MyD88, but also possess its own pathways upon MALP-2 stimulation [46]. To investigate the role of Mal in MALP-2-induced HO-1 expression and to compare the role of MyD88 and Mal in mediating HO-1 expression, we transfected cells expressing DN-Mal plasmid or Mal siRNA, and performed HO-1 luciferase activity assay. The results showed that the HO-1 expression level was significantly decreased when the cells were transfected with DN-Mal plasmid (Fig. 5A, Fig. S5). In addition, MALP-2-stimulated HO-1 expression was significantly reduced when Mal was silenced by siRNA (Fig. 5B). Although both of DN-MyD88 and DN-Mal decreased the promoter activity of HO-1 to some degree, but the luciferase activity in cells transfected with DN-Mal was much lower than that in MyD88 mutant cells (Fig. 5C). Taken together, these data indicated that Mal played a greater role than MyD88 in HO-1 induction by MALP-2.

### PI3K is involved in MALP-2-induced HO-1 expression

**Figure 6:**
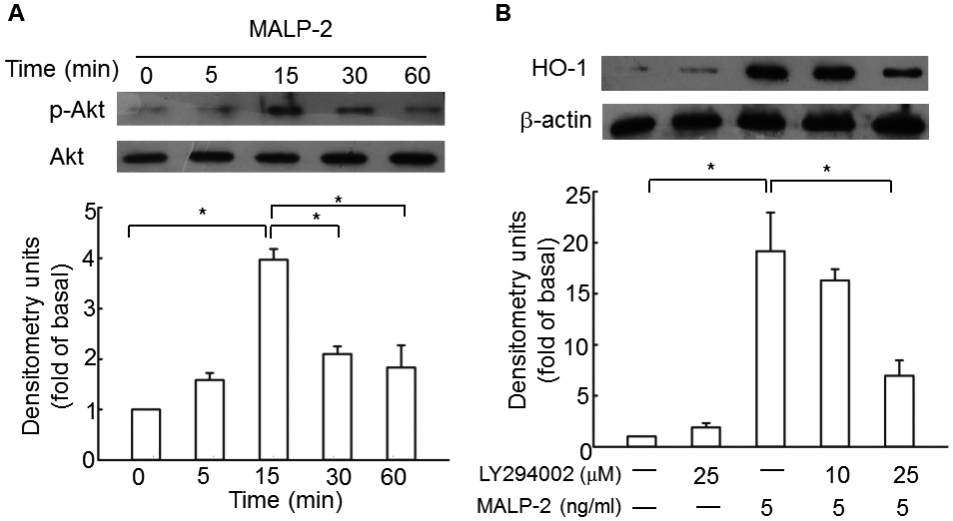
Involvement of PI3K in the up-regulation of HO-1 by MALP-2. **A.** Cells were stimulated with MALP-2 for the indicated time intervals, the cell lysates were subjected to Western blotting using an anti-p-Akt antibody. **B.** THP-1 cells were pretreated with LY294002 (25 µM) for 1 h, and then incubated with 5 ng/ml MALP-2 for another 16 h. Densitometric analysis was performed after normalization with total Akt or β-actin. All of the values are expressed as means ± SEM of three independent experiments. *, P<0.05 for comparisons within groups.

The PI3Ks are a family of lipid kinases involved in a broad range of cellular responses from cell cycle regulation, apoptosis, growth, and cell survival. PI3Ks can be divided into 3 main classes on the basis of their in vitro lipid substrate specificity. Class 1A PI3Ks are heterodimers comprising a regulatory subunit (p85) and a catalytic subunit (p110) and are activated downstream of receptors involved in protein tyrosine kinase signaling [47]. Activated PI3K catalyses PIP2 to PIP3, which, in turn, binds to and phosphorylates Akt through the phosphatidylinositol-dependent kinases [48]. To test whether the activation of PI3K was involved in MALP-2-induced HO-1 expression, a PI3K inhibitor, LY294002, was used and Akt phosphorylation was used as a readout of PI3K activity. The results showed that MALP-2 induced phosphorylation of Akt at 5 min, and the peak was reached at 15 min (Fig. 6A, Fig. S6). MALP-2-induced HO-1 expression was abrogated by pretreatment with 25 µM LY294002 (Fig. 6B). These data demonstrated that PI3K played critical roles in MALP-2-induced HO-1 expression.

### Btk and c-Src are associated with Mal and regulate PI3K activation induced by MALP-2

**Figure 7:**
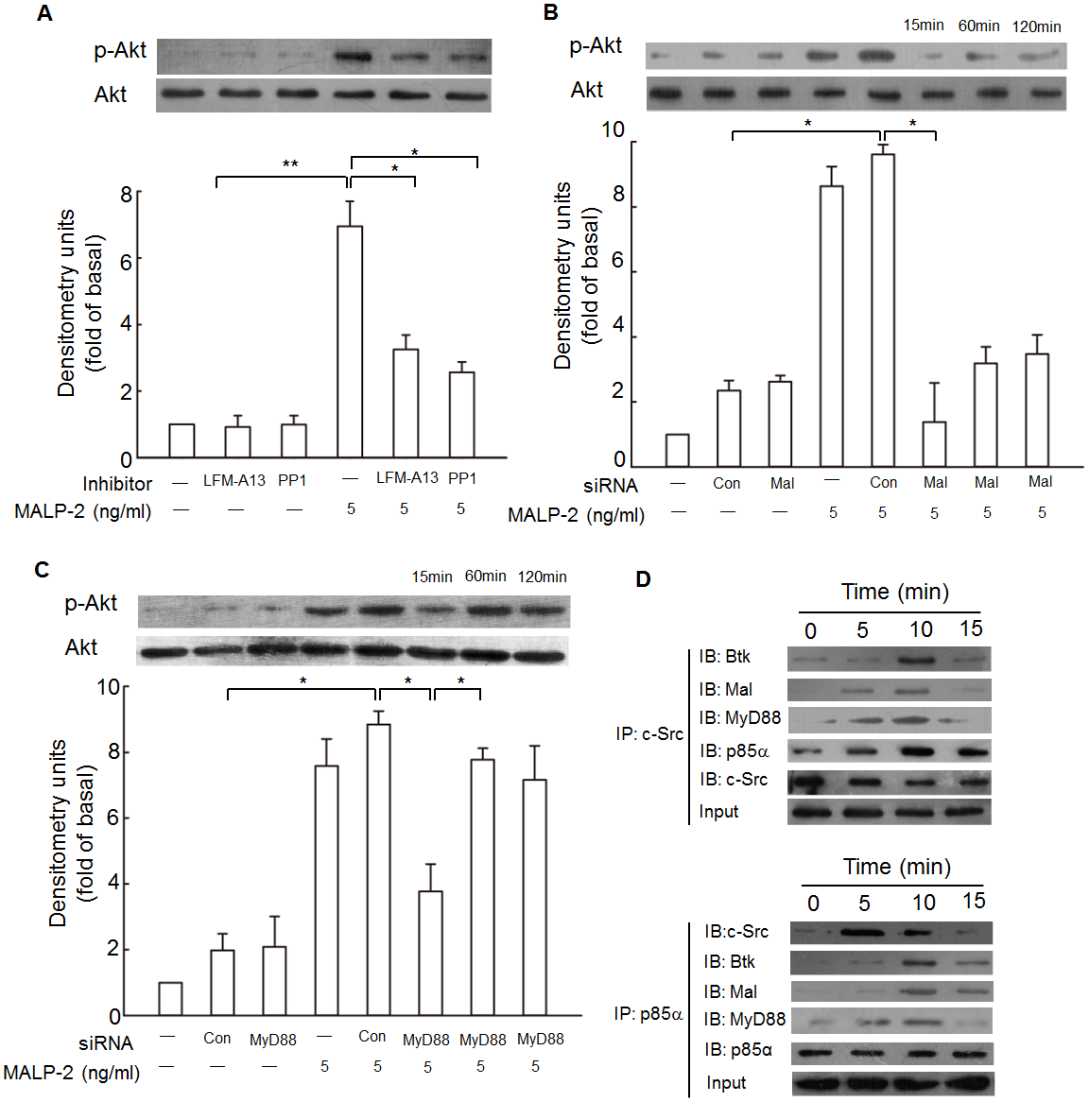
MALP-2 induces PI3K activation is regulated by the formation of a Btk, Mal, c-Src and p85α complex. **A.** Cells were pretreated with or without LFM-A13 (100 µM), or PP1 (50 µM) for 1 h, and then incubated in the absence or presence of 5 ng/ml MALP-2 for 15 min. Cell fractions were prepared and subjected to Western blotting analysis with an anti-p-Akt antibody. Data shown represents three experiments (means ± SEM). *, P<0.05 and **, P<0.01 for significant difference between compared groups. **B, C.** Cells were transfected with siRNA of Mal or MyD88, and then treated with MALP-2 for 15, 60 or 120 min. Phosphorylated Akt was detected by Western blotting. *, P<0.05 for significant difference between compared groups. **D.** Cells were treated with MALP-2 for the indicated times, the cell lysates were subjected to immunoprecipitation using an anti-c-Src or anti-p85α antibody, and followed by immunoblotting with anti-Btk, anti-Mal, anti-MyD88, anti-c-Src or anti p85α antibody. All figures are representative of three independent experiments.

Non-receptor tyrosine kinase, such as Btk and c-Src, have been shown to mediate PI3K activation [49]. To investigate whether Btk and c-Src were involved in Akt phosphorylation and PI3K activation was mediated by Mal or MyD88, and to further observe the physical interactions between Btk, c-Src, Mal, MyD88 and PI3K in response to MALP-2, LFM-A13 and PP1 were used to observe the status of phosphorylated Akt, and THP-1 cells after MALP-2 stimulation were lysed and subjected to immunoprecipitation using anti-Btk, anti-Mal, anti-MyD88, anti-c-Src or anti-p85α antibody. The results showed that MALP-2-induced phosphorylation of Akt was suppressed by pretreatment with PP1 and LFM-A13 (Fig. 7A, Fig. S7). In addition, transfection with siRNA of Mal severely impaired Akt phosphorylation after stimulation by MALP-2 (Fig. 7B). In contrast, Akt phosphorylation in MyD88 siRNA-transfected cells was significantly decreased at 15 min after stimulation. Interestingly, as stimulation continues, Akt phosphorylation reoccurred after about 60 min and lasted until 120 min (Fig. 7C). The protein levels of Btk, Mal, MyD88 and p85α were increased in a time-dependent manner in c-Src immunoprecipatated complex. Moreover, the cell lysates immunoprecipitated using a p85α antibody also enhanced the association among c-Src, Btk, Mal and MyD88 in response to MALP-2 (Fig. 7C). These results suggested that Btk and c-Src were associated with Mal and regulated PI3K activation induced by MALP-2.

### PI3K mediates MALP-2-induced Nrf2 activation

**Figure 8:**
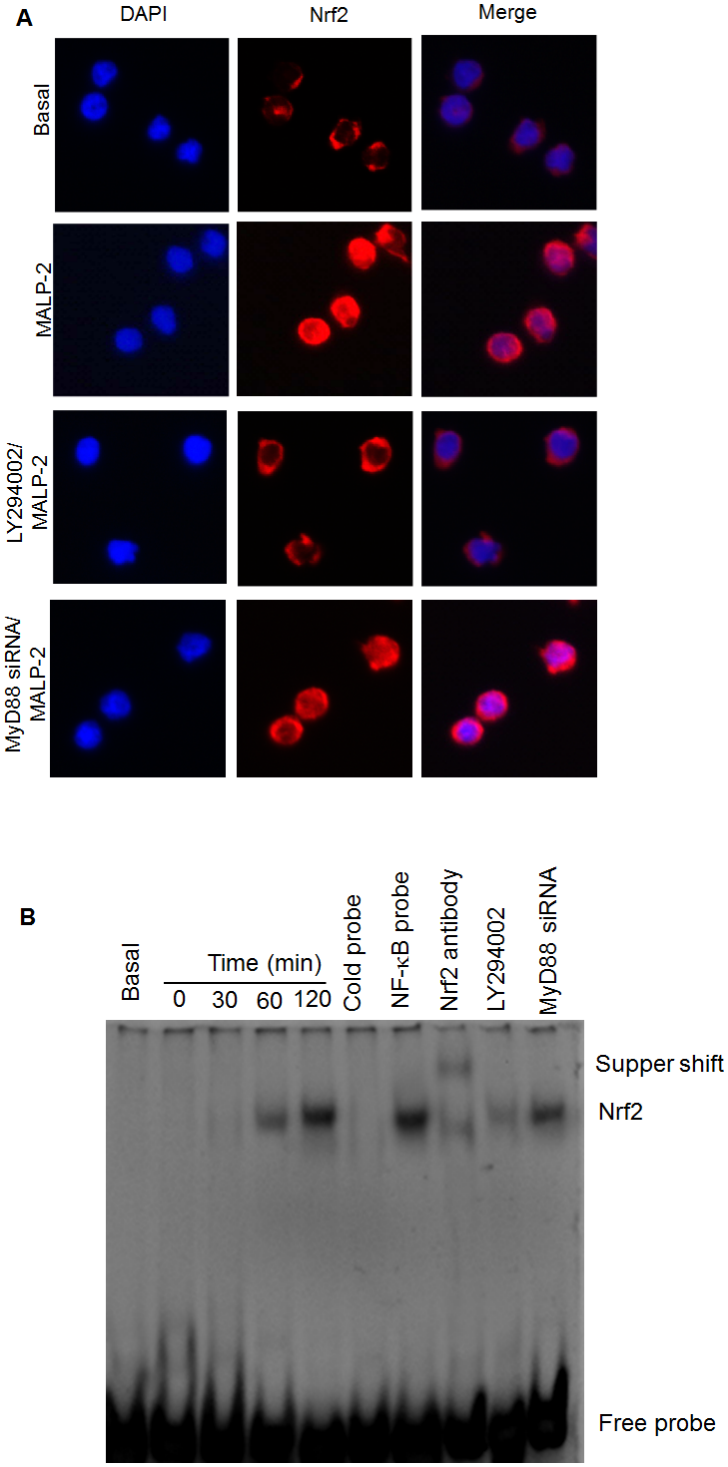
MALP-2 stimulates translocation of Nrf2 to the nucleus and binding to HO-1 ARE in THP-1 cells. **A.** Immunofluorescence images of THP-1 cells pretreated with or without LY294002 (25 µM) and MyD88 siRNA in the presence of MALP-2 (5 ng/ml) for 2 h. Cells were fixed and then stained with 4′-6-diamidino-2-phenylindole or anti-Nrf2 antibody, followed by incubation with cy3-conjugated anti-rabbit antibody. Images were obtained using a confocal microscope. **B.** EMSA was performed using MALP-2-stimulated nuclear THP-1 extracts and biotinylated HO-1-ARE probe. The specificity of Nrf2 was verified by competition analysis with an excess of nonlabeled specific (cold probe) or nonspecific (NF-κB probe) oligonucleotide probe. For supershift analysis, nuclear extracts from MALP-2-treated THP-1 cells were preincubated with 1 µg anti-Nrf2 supershift antibody before EMSA.

It is well-known that the redox-sensitive basic leucine zipper transcription factor Nrf2 regulates a variety of antioxidant response element (ARE)-dependent gene transcription [25]. To investigate whether PI3K was involved in Nrf2 activation, we used immunofluorescence and EMSA approaches. Immunofluorescence results showed that treatment with MALP-2 led to marked increase of the Nrf2 immunofluorescence signal in the nucleus, suggesting a strong translocation of Nrf2 from the cytoplasma into the nuclear areas in MALP-2-treated monocytes (Fig. 8A, Fig. S8). When the cells were pretreated with LY294002, nuclear translocation of Nrf2 was significantly attenuated (Fig. 8A). In contrast, nuclear translocation of Nrf2 was almost not affected by cells transfected with MyD88 siRNA. Furthermore, EMSA results showed that Nrf2 binding to the ARE promoter sequence appeared at 1 h and continued to increase until 2 h, this binding of the complex could be specifically inhibited by the addition of unlabeled oligonucleotide, and an antibody directed against Nrf2 retarded the migration of the complex, indicating the presence of Nrf2 in the ARE-nuclear protein complex. Similarly, the Nrf2-ARE-binding complex was inhibited by treatment with LY294002. In contrast, the complex was only partially inhibited in MyD88 siRNA-transfected cells (Fig. 8B). These data indicated that MALP-2-induced Nrf2 activation was mediated by PI3K.

## Discussion

Membrane lipoproteins have been shown to play an important role in mycoplasmal diseases that are associated with inflammation [19]. However, extraction of membrane lipoproteins from mycoplasmas is not easy because of contamination by endotoxin. MALP-2 is a synthesized lipopeptide based on the general structure of membrane lipoproteins and shares most of the immune-stimulating activity in many inflammatory gene expression [11]. HO-1 is the rate-limiting enzyme that catalyzes the degradation of heme into CO, Fe^2+^ and bilirubin [50]. Recently, HO-1 has been reported to be induced in the autoregulation that prevents an excessive activation of macrophages after TLR stimulation [29]. In our previous study, we showed that MALP-2 induced HO-1 expression in monocytes [31]. However, the elaborate regulatory mechanism remains unclear. The present work provides three major findings. First, MyD88 is not necessary for the induction of HO-1 in response to MALP-2 stimulation, and in contrast, Mal plays a greater role in mediating HO-1 expression. Second, the association of Btk, c-Src and Mal is required for MALP-2-triggered signaling for the activation of PI3K. Third, c-Src, Btk, Mal and MyD88 form a physical complex to activate PI3K, which further induces Nrf2 translocation to the nucleus and binds to the ARE-site to stimulate HO-1 expression.

Monocytes and macrophages are the major professional phagocytes in the eradication of the invading mycoplasmas. In mammals, the innate immune system can recognize the mycoplasma membrane lipoproteins by TLR2 and TLR6. Binding of the lipoproteins induces a conformational change of the TLR2/6 dimer, activates signaling mediators including IRAKs, TRAF6 and MAPKs, ultimately leads to the activation of specific inflammatory nuclear transcription factors and initiates inflammatory responses [12]. This innate immune response constitutes to the first line of defense against mycoplasma. However, sustained inflammatory signaling can be toxic and induce the pathogenesis of autoimmune, chronic inflammatory and infectious diseases. Therefore, the intensity and duration of TLR response must be tightly controlled. Our study clearly demonstrated that MALP-2 induced expression of HO-1 in THP-1 cells through TLR2 or TLR6, as shown by pretreatment with anti-TLR2 and TLR6 neutralizing antibodies. This hypothesis was further supported by the transfection with dominant negative mutant of TLR2 or TLR6. Together with the importance of HO-1 in inflammatory response, these results suggested that expression of HO-1 may be an adaptive negative-feedback mechanism in order to synchronize the positive activation and negative regulation of TLR signal transduction to avert potentially harmful immunological consequences. However, when and to what extent MALP-2 could initiate a protective signal is still unknown. It seems that initial exposure to low doses of MALP-2 might sensitize TLR2 signaling, which may be necessary to begin protective immune responses that may prevent bacterial growth and subsequent immunopathology [35]. As stimulus continues, increased inflammatory mediators (reactive oxygen species, TNF-α, prostaglandins and derivatives) might activate NADPH oxidase and Nrf2 to control the monocytes and macrophages. Recently, Atuart et al. found that TNF induced TNF up-regulation by an autocrine mechanism to modulate prolonged Nrf2-induced gene expression, to protect against inflammatory impairment [26].

One novel finding in our work is that MALP-2-mediated HO-1 expression was largely dependent on Mal. Previous works on the role of Mal in recruiting MyD88 to the plasma membrane in TLR signaling suggested the bridging role of Mal in TLR2 and TLR4 signaling to the MyD88-dependent pathway. This is supported by the fact that the TIR-domain surface of MyD88 is electropositive, and the surface of Mal is predicted to be electronegative [45]. However, Kenny et al. demonstrated that at high concentrations of TLR1/2 ligand or MALP-2, the induction of IL-6 and activation of NF-κB were independent of Mal, but at low dose of these ligands, Mal might sensitize TLR2 signaling [35]. Another extensive study demonstrated that Mal was located upstream of MyD88 in MALP-2-induced signaling and was TLR2-dependent, but TLR6-independent signaling was independent of Mal [40]. Here, we have taken an indepth look at the role of MyD88 and Mal in mediating HO-1 expression. Surprisingly, in contrast to the universal role of MyD88, it only displayed a subtle function in MALP-2-induced HO-1 expression, as demonstrated by the transfection with a dominant negative mutant of MyD88 or siRNA. Accordingly, silencing of Mal severely blocked most of the induction. Requirement of Mal in MALP-2-induced HO-1 production was further backed by the construction of HO-1 luciferase plasmid. Although the induction of luciferase transcription was reduced in MyD88 mutant cells, it was still higher than that of cells transfected with dominant negative Mal plasmid. Therefore, Mal has distinct functional roles that may depend on the TLR heterodimers and the concentration of ligands. Our observations support the model in which Mal is the adapter protein in utmost proximity to TLR2/6 in MALP-2-mediated HO-1 expression while MyD88 is somewhat dispensable. To the best of our knowledge, this is the first report to demonstrate that Mal is involved in the expression of stress-inducible proteins to regulate cellular defense. As a matter of fact, it was reported that Mal-mediated negative regulation of inflammatory response could be degraded by binding to the SH2 domain of the suppressor of cytokine signaling 1 (SOCS-1), or by phosphorylation induced by IRAK1 and IRAK4 [51], [52]. However, the concept that Mal down-regulates inflammation needs further investigation.

Another finding of the current report is that non-receptor tyrosine kinases are involved in MALP-2-induced HO-1 expression. Our study suggested that Btk served as a modulator for stress-inducible genes, which concurred with earlier reports in which Btk was involved in the induction of HO-1 by lipoteichoic acids and lipopolysaccharides [29]. In an earlier report, Honda et al. showed that Btk is a negative regulator of signal transduction that leads to the activation of NADPH oxidase and a molecule that prevents excessive neutrophil response [53]. However, the mechanism of Btk in TLR pathways remains to be elucidated. Phosphorylation of Mal by Btk is an important step [33], during which both the pleckstrin homology and kinase domains of Btk were necessary for the association with Mal and served as negative regulators of the TLR pathways. Btk-deficient neutrophils exhibited higher production of reactive oxygen species and were associated with more apoptosis [53]. c-Src is another important tyrosine kinase implicated in innate immune response and signaling induced by cytokines. In the present study, MALP-2 induced c-Src phosphorylation. Activated c-Src could phosphorylate downstream kinases, such as Btk, to mediate HO-1 expression, as demonstrated by pretreatment with PP1 or by transfection with c-Src siRNA in the present study.

In addition to the activation of Btk and c-Src, PI3K was also activated upon MALP-2 stimulation, as indirectly measured by the phosphorylation of Akt, a downstream component of PI3K. LY294002 treatment inhibited MALP-2-induced HO-1 expression. Involvement of Btk, Mal, c-Src and PI3K in MALP-2-induced HO-1 expression suggested us to test whether PI3K was linked to Btk, Mal and c-Src. The first evidence came from the application of inhibitors for Btk and c-Src. By using LFM-A13 and PP1, we detected decreased phosphorylation of Akt, indicating that MALP-2-induced PI3K activation was mediated by Btk and c-Src. Next, we found that mutant of Mal almost completely blocked most of Akt phosphorylation. Although MyD88 plays subtle role in contrast to Mal in HO-1 expression, but at this stage, the positive role of MyD88 in mediating PI3K activation cannot be neglected. A former study demonstrated that LTA-induced HO-1 expression was mediated by TLR2, MyD88 and ROS-dependent Nrf2 activation [24]. In our study, we found that MALP-2-induced Akt phosphorylation was only reduced but not diminished in MyD88-silenced THP-1 cells, which was consistent with the results discovered previously that the PI3K activity in response to TLR2/6 agonists was delayed in MyD88-deficient macrophages [40]. It seems that under MALP-2 stimulation, the presence of MyD88 may mediate the rapid formation of a signaling platform that increases the affinity of docking sites for other signaling molecules, such as PI3K to close proximity with Mal for interaction. As expected, we detected a physical interaction between c-Src, Btk, Mal, MyD88 and PI3K according to immunoprecipitation assay. The detailed protein-protein interactions among c-Src, Btk, Mal, and PI3K are still unknown. Although TLR2 and TLR6 contain the binding sites for PI3K, but they are not likely to interact with each other in this manner. The interaction of Mal with PI3K p85α is specific and does not require TLR2 as an intermediate, since MALP-2 does not drive Akt phosphorylation in Mal-deficient cells [54]. Based on the above results, we still cannot disregard the possibility that c-Src or Btk could bind PI3K directly, because PI3K could directly interact with Btk via SH3-domain after LTA treatment [49]. However, it is less likely that direct interactions between c-Src or Btk and PI3K could occur after MALP-2 stimulation, as long as MALP-2 does not promote Akt phosphorylation in Mal-deficient cells [54]. Mal binds to the site of high substrate phosphatidylinositol 4,5-bisphosphate through a specific amino-terminal domain [40]. On the other hand, Mal contains a TRAF6-binding domain, which can lead to NF-κB activation in response of TLR2 stimulation [55]. However, the interaction of Mal with PI3K is privileged over that between Mal and TRAF6, since Mal-dependent activation of NF-κB is dependent on MyD88 [40].

Activation of PI3K regulates many biological processes, including Nrf2, a key factor in ARE-mediated gene induction of antioxidant proteins in response to various stimuli [41]. Our results clearly showed that MALP-2 stimulation could significantly induce Nrf2 translocation to the nucleus, and its binding to the cis-acting ARE to induce HO-1 expression. Furthermore, we also found that PI3K was involved in MALP-2-induced Nrf2 translocation, as it could be blocked by LY294002, while MyD88 seems somewhat dispensable.

**Figure 9:**
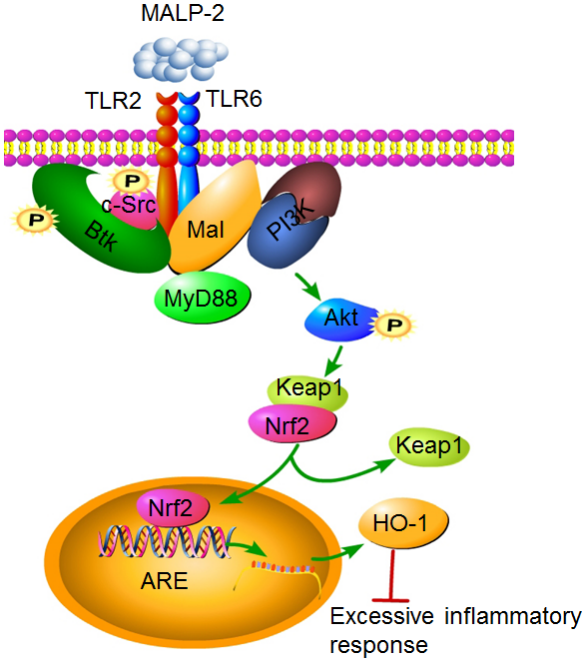
Schematic model depicting the potential signaling pathway involved in MALP-2-induced HO-1 expression in THP-1 cells. The cytoplasmic adaptor Mal recruits the TLR2/6 heterodimer in response to MALP-2 stimulation. Mal binds to p85α, the regulatory subunit of PI3K, in the presence of Btk and c-Src. PI3K activates downstream events like Akt phosphorylation and Nrf2 nuclear translocation, which in turn initiates HO-1 expression.

In summary, our present study supports a model in which Mal not only aids MyD88 to the membrane, but also exhibits its own pathway to activate PI3K by forming a c-Src, Btk, Mal, MyD88 and PI3K complex in response to MALP-2 stimulation (Fig. 9). Activated PI3K could induce Nrf2 recruit to the nucleus and binding to the promoter region of HO-1, leading to up-regulated HO-1 expression. HO-1 induction may prevent excessive activation of monocytes/macrophages, or form a feedback loop that negatively regulates the inflammatory response in the host.

## Supporting Information

Figure S1 **Additional western blots.**
(TIF)

Figure S2 **Additional western blots.**
(TIF)

Figure S3 **Additional western blots.**
(TIF)

Figure S4 **Additional western blots.**
(TIF)

Figure S5 **Additional western blots.**
(TIF)

Figure S6 **Additional western blots.**
(TIF)

Figure S7 **Additional western blots.**
(TIF)

Figure S8 **Immunofluorescence images.**
(TIF)
