# Supplementary figures and images for: Macrophage-Activating Lipopeptide-2 Requires Mal and PI3K for Efficient Induction of Heme Oxygenase-1
Source: PLoS One. 2014 Jul 31;9(7):e103433. doi: 10.1371/journal.pone.0103433 (PMC4117634; doi:10.1371/journal.pone.0103433)

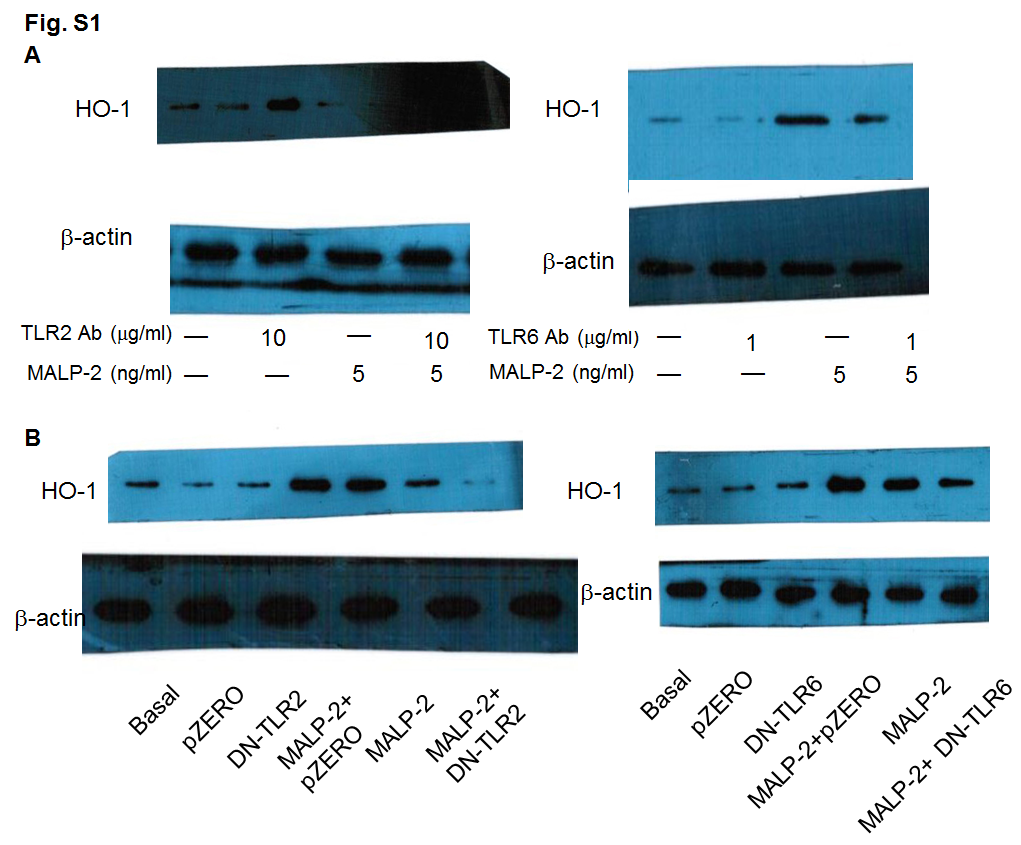

Supplement: Figure S1 — Additional western blots. (TIF) [file pone.0103433.s001.tif]

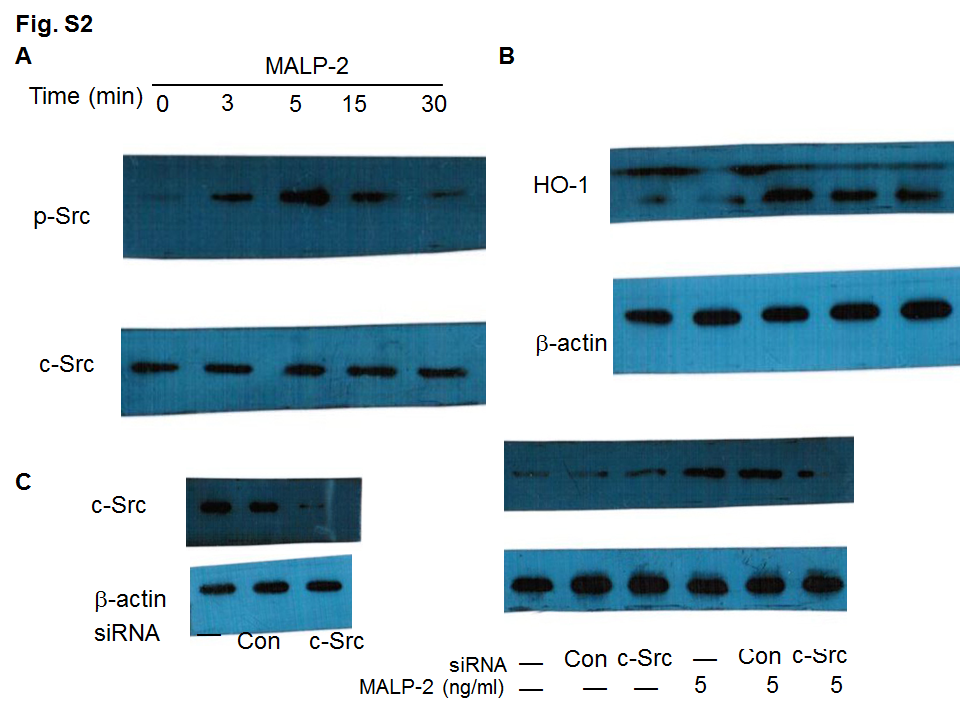

Supplement: Figure S2 — Additional western blots. (TIF) [file pone.0103433.s002.tif]

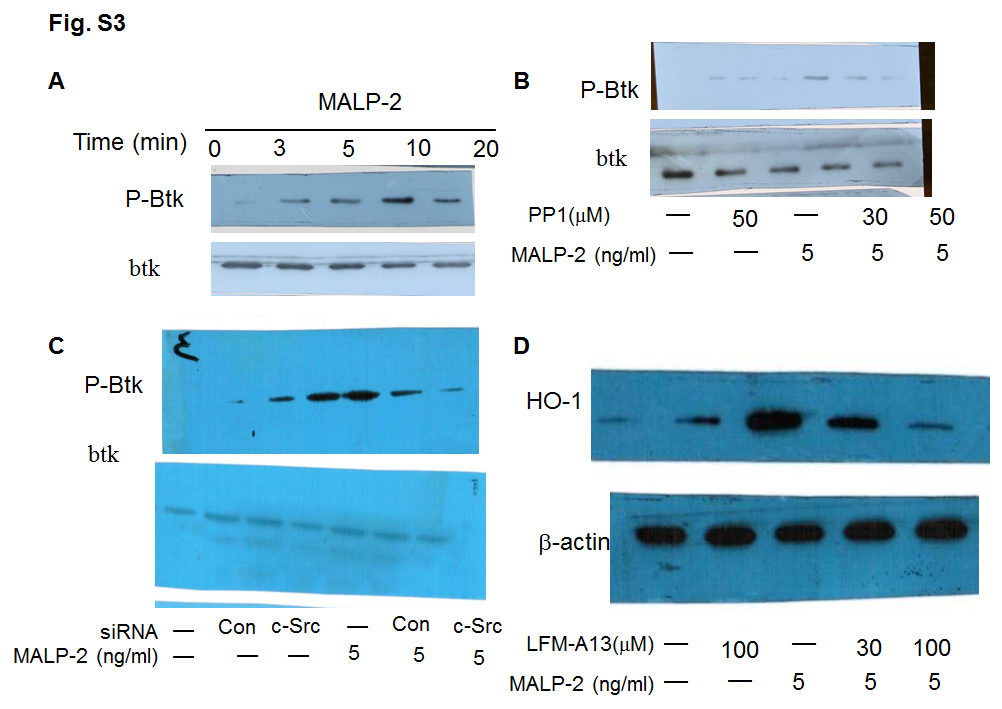

Supplement: Figure S3 — Additional western blots. (TIF) [file pone.0103433.s003.tif]

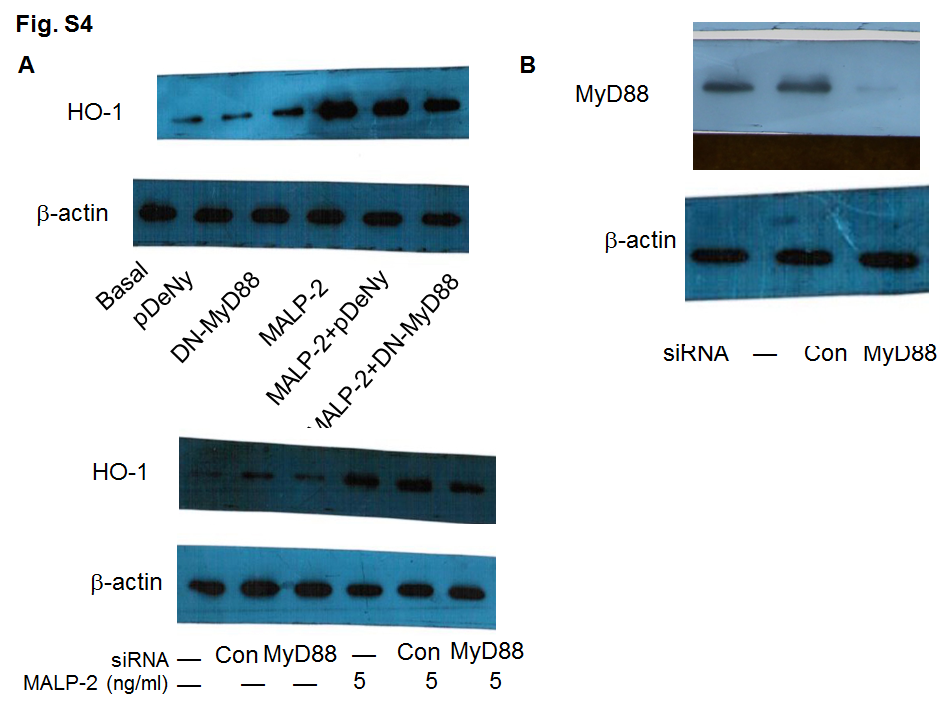

Supplement: Figure S4 — Additional western blots. (TIF) [file pone.0103433.s004.tif]

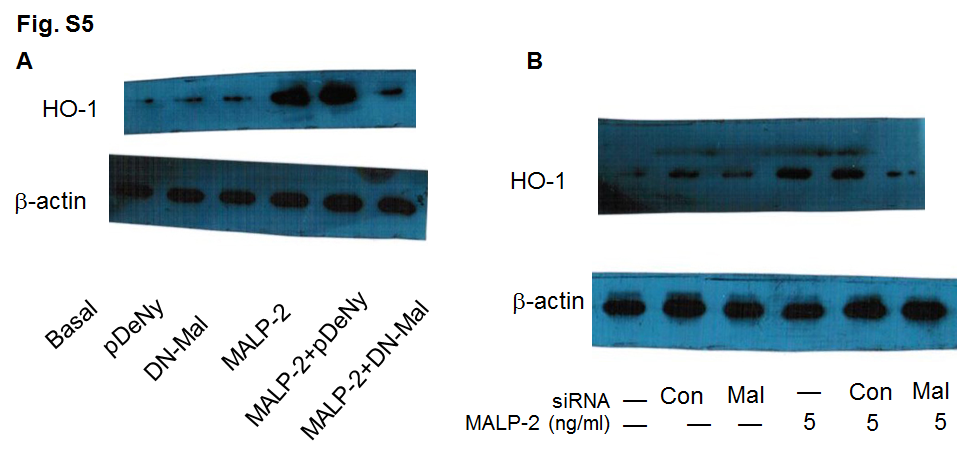

Supplement: Figure S5 — Additional western blots. (TIF) [file pone.0103433.s005.tif]

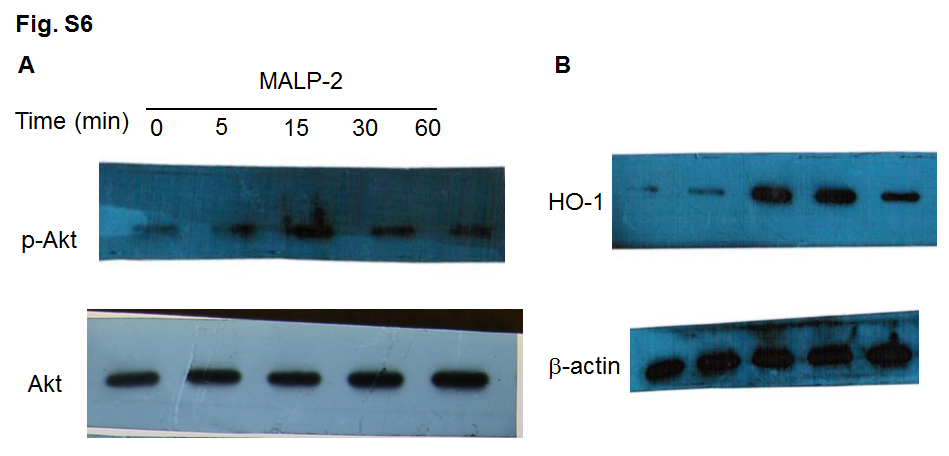

Supplement: Figure S6 — Additional western blots. (TIF) [file pone.0103433.s006.tif]

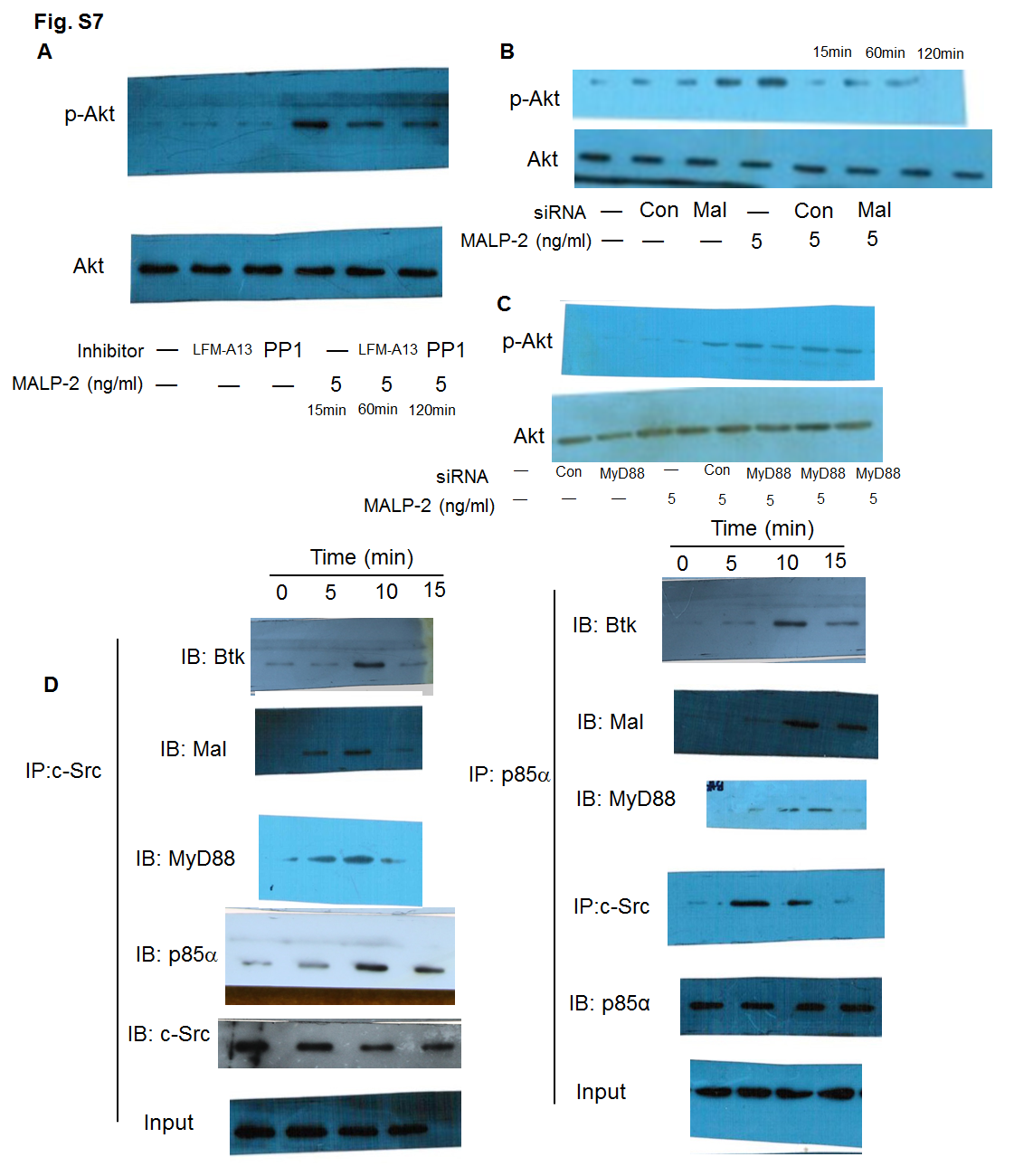

Supplement: Figure S7 — Additional western blots. (TIF) [file pone.0103433.s007.tif]

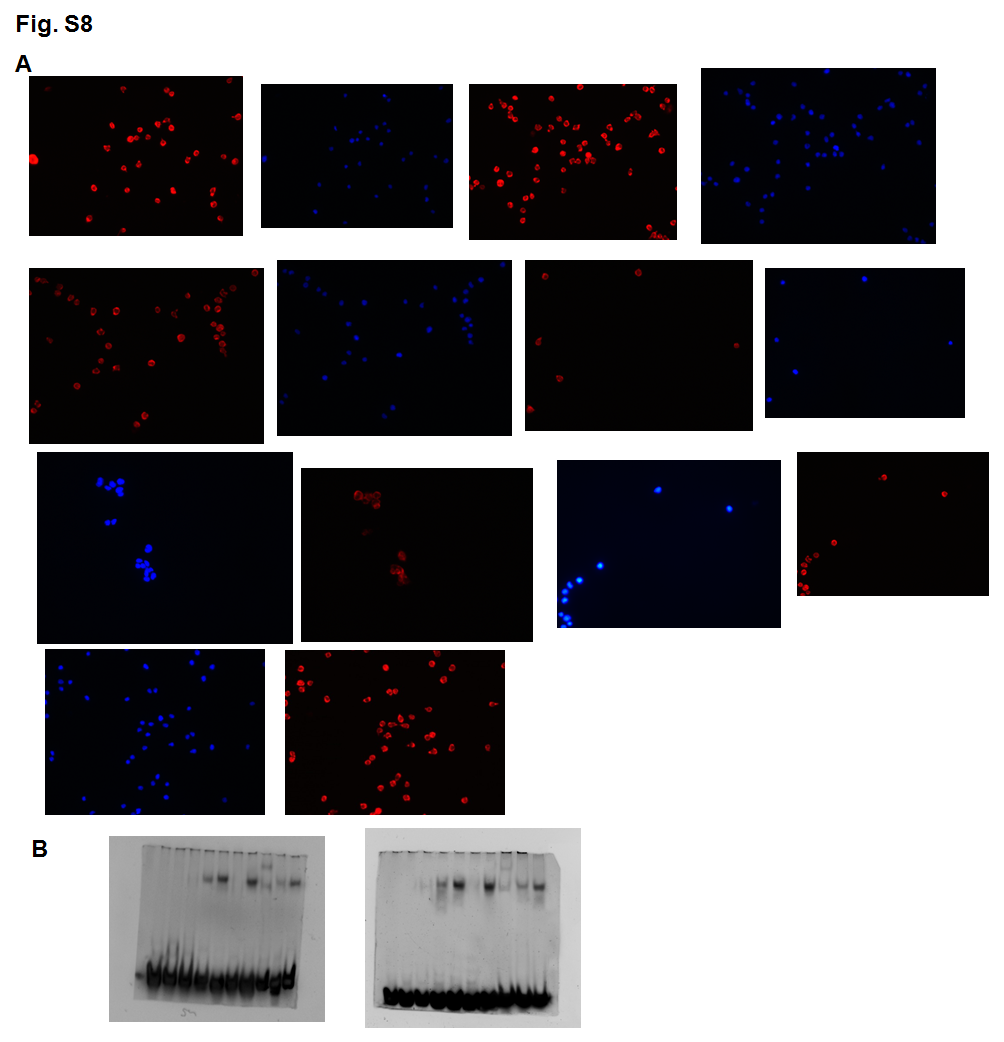

Supplement: Figure S8 — Immunofluorescence images. (TIF) [file pone.0103433.s008.tif]
